# Clinical Aspects and Disease Severity of *Streptococcus dysgalactiae* Subspecies *equisimilis* Bacteremia, Finland^1^

**DOI:** 10.3201/eid3009.240278

**Published:** 2024-09

**Authors:** Viivi Nevanlinna, Janne Aittoniemi, Reetta Huttunen, Tiina Luukkaala, Sari Rantala

**Affiliations:** Tampere University, Tampere, Finland (V. Nevanlinna, R. Huttunen, T. Luukkaala, S. Rantala);; Tampere University Hospital, Tampere (V. Nevanlinna, R. Huttunen, T. Luukkaala, S. Rantala);; Fimlab Laboratories, Tampere (J. Aittoniemi)

**Keywords:** *Streptococcus dysgalactiae* subspecies *equisimilis*, *Streptococcus dysgalactiae*, SDSE, group G *Streptococcus*, bacteremia, disease severity, intensive care unit, Pitt bacteremia score, group C* Streptococcus*, bacteria, streptococci, Finland

## Abstract

We conducted a prospective study of 159 cases of *Streptococcus dysgalactiae* subspecies *equisimilis* (SDSE) bacteremia in 157 patients at 2 hospitals in Finland during November 2015–November 2019. Cellulitis was associated with nonsevere disease (p = 0.008); necrotizing fasciitis was associated with severe disease (p = 0.004). Fifty percent of patients had >1 clinical characteristic associated with risk for death. The case-fatality rate was 6%, and 7% of patients were treated in an intensive care unit. Blood leukocyte counts on days 2 (p = 0.032) and 3 (p = 0.020) and C-reactive protein levels on days 3 (p = 0.030) and 4 (p = 0.009) after admission were predictors of severe disease. The Pitt bacteremia score was an accurate predictor of death. Using the Pitt bacteremia score, leukocyte counts, and CRP responses during initial treatment can improve treatment strategies and survival for patients with SDSE.

*Streptococcus dysgalactiae* subspecies *equsimilis* (SDSE) is a β-hemolytic *Streptococcus* species mainly expressing Lancefield antigens C or G (*1*). The incidence of SDSE bacteremia has been increasing, and SDSE has surpassed *S*. *pyogenes* as the primary cause of β-hemolytic streptococcal bacteremia in several countries (*2*–*4*).

The clinical manifestations of SDSE bacteremia resemble those caused by *S*.* pyogenes*, such as cellulitis, pneumonia, endocarditis, and necrotizing fasciitis (*5*). Death associated with SDSE bacteremia has varied from 8% to 20% (*6*–*9*), reaching 33% in patients with more severe clinical manifestations (*10*). Nevertheless, only a few studies provide data on disease severity of SDSE bacteremia (*8*,*11*–*15*), and information on factors contributing to severe disease is lacking. In a study from Canada, 70% of SDSE bacteremia episodes were associated with severe disease, involving admission to an intensive care unit (ICU) or the need for vasopressor or ventilator support (*11*).

Endocarditis has been observed in 6%–7% of SDSE bacteremia patients; 29% of those patients required ICU treatment, and embolic complications developed in 45%–46% (*11*,*16*,*17*). Although necrotizing fasciitis has been relatively uncommon among SDSE bacteremia patients, a large multicenter study on necrotizing soft tissue infections identified SDSE as the second most common causative agent after *S*.* pyogenes*, contributing to 7% of cases (*18*). Septic shock developed in 41% of patients with necrotizing soft tissue infections caused by SDSE, and 89% of those required mechanical ventilation (*18*).

The Pitt bacteremia score (PBS) is a clinical scoring system used to assess the severity of acute illness in patients with bloodstream infections (*19*). PBS uses a scale ranging from 0 to 14 points; patients with a PBS score >4 are categorized as critically ill and have a high death risk. Compared with other scores that determine acute severity of illness, PBS has performed well and is simple to use because it is solely derived from variables identified during the initial physical examination. PBS has been extensively assessed in bloodstream infections and also in nonbacteremic infections; however, the accuracy of the score has not been evaluated for SDSE bacteremia (*19*–*21*).

We conducted a prospective study on clinical aspects and disease severity in patients with SDSE bacteremia. The aim of this study was to examine clinical manifestations of the disease, antibiotic treatment and susceptibility, laboratory test results associated with severe disease, and clinical manifestations requiring ICU treatment. Furthermore, we used the Pitt bacteremia score to assess severity of acute illness in SDSE bacteremia.

## Methods

Tampere University Hospital (TAUH) in Tampere, Finland, is the second-largest tertiary-care hospital in Finland, serving a catchment population of ≈535,000 residents within the Pirkanmaa health district. We included adult patients with >1 SDSE-positive blood culture who were hospitalized at TAUH or Hatanpää City Hospital in Tampere during November 2015–November 2019 in the study population. At the beginning of 2017, Hatanpää City Hospital was integrated with TAUH. During the study period, we excluded 3 cases of *S*.* canis* infection, and 2 patients declined to participate. Consequently, the final study population consisted of 159 episodes involving 157 patients with SDSE bacteremia. The study was approved by the Regional Ethics Committee of Tampere University Hospital.

Blood samples were collected in the emergency room at Tampere University Hospital and were studied and cultivated at Fimlab Laboratories in Tampere. During November 2015–October 2017, blood samples were collected into ﻿BacT/Alert FA Plus aerobic and FN Plus anaerobic blood culture bottles and incubated in an automated BacT/Alert ﻿3D microbial detection system ﻿(bioMérieux). During November 2017–November 2019, blood samples were collected in BD BACTEC Plus ﻿Aerobic/F and Lytic/10 Anaerobic/F culture vials and ﻿incubated in a BD BACTEC FX blood culture ﻿system (Becton Dickinson). 

﻿We identified SDSE primarily on the basis of typical large colony-forming growth and β-hemolysis on blood agar plates. Until February 2017, we identified bacteria by using latex bead agglutination to determine Lancefield grouping (PathoDxtra Strep Grouping Kit; Thermo Fisher Scientific) and confirmed identity by using API 20 Strep (bioMérieux) or matrix-assisted laser desorption/ionization time-of-flight (MALDI-TOF) mass spectrometry (VITEK MS instrument; bioMérieux). Since March 2017, MALDI-TOF mass spectrometry has been the primary method for identification. MALDI-TOF analysis provided results for *S. dysgalactiae* subsp. *dysgalactiae/equisimilis*, which was interpreted as *S. dysgalactiae* subsp. *equisimilis* associated with human disease.

During the study period, a clinical microbiologist (J.A. or T.S.) contacted an infectious disease specialist (S.R.) regarding all SDSE-positive blood cultures. Concurrently, the specialist kept track of SDSE-positive blood cultures in Finland’s register for hospital infections and antimicrobial drug use, where all positive blood cultures in Finland are recorded, and contacted SDSE bacteremia patients to obtain informed consent for study participation. In cases where the patient was unable to provide informed consent because of deteriorating condition, we obtained informed consent from the patient’s first-degree relatives. We gathered clinical data through a combination of patient interviews and examinations. In addition, we conducted a review of the patients’ medical records both during and after hospitalization.

The variables included in the PBS were measured at hospital admission, and we calculated the score for each patient. We defined hypotension as a systolic blood pressure of <90 mm Hg or the need for vasopressors. Mechanical ventilation refers to the use of invasive mechanical support. We categorized mental status as disoriented or unconscious upon admission. Because no specific data on unconsciousness existed at admission, we considered a patient to be unconscious if their mental status deteriorated after admission and the patient was recorded as unconscious during the first 2 days of hospitalization. Body temperature was measured at hospital admission. Data on cardiac arrests were unavailable and were excluded from the score.

We analyzed clinical manifestations and laboratory test results in relation to severe disease, which we defined as treatment in ICU or death within 30 days after hospital admission. We analyzed categorical data by using the χ^2^ or Fisher exact test, as appropriate, and analyzed nonparametric data by using the Mann-Whitney U test. We evaluated whether laboratory test results and the PBS predicted death by using receiver operating characteristic (ROC) curves (*22*). For the ROC method, the area under the curve (AUC) is 1.0 if both sensitivity and specificity of the test are 100% and 0.5 if the test has no diagnostic value. We used the Youden index (sensitivity + specificity – 1) to determine optimal cutoff levels for PBS and laboratory test results that had statistically significant AUCs predicting severe disease or death. We calculated odds ratios (ORs) and 95% CIs and considered p values of <0.05 statistically significant. We performed all statistical analyses by using SPSS Statistics for Mac version 29 (IBM).

## Results

The study comprised 159 SDSE bacteremia episodes in 157 patients during November 2015–November 2019. The median patient age was 71 (range 28–93) years, and 95% of patients had underlying conditions. Sixty percent of patients were male and 40% female; 71% percent of patients with severe disease were male and 29% female. The most common clinical manifestations in all patients were cellulitis (69%), purulent skin infections (19%), and pneumonia (15%) (Table 1). Cellulitis was associated with nonsevere disease (p = 0.008), whereas necrotizing fasciitis was associated with severe disease (p = 0.004). Endocarditis was diagnosed in 5 patients, but none of those patients experienced embolic complications, died, or needed intensive care or surgical treatment.

**Table 1:** Clinical manifestations and disease severity in patients with *Streptococcus dysgalactiae* subspecies *equisimilis* bacteremia during November 2015–November 2019, Finland*

Infection type†	Nonsevere disease, n = 142	Severe disease, n = 17	p value
Skin/soft tissue infection	107 (75)	10 (59)	0.154
Cellulitis	103 (73)	7 (41)	0.008
Purulent skin infection	27 (19)	4 (24)	0.746
Necrotizing fasciitis	1 (1)	3 (18)	0.004
Deep abscess	13 (9)	2 (12)	0.664
Bursitis	2 (1)	0	NA
Bone and joint infection	16 (11)	2 (12)	1.000
Osteomyelitis, all	7 (5)	1 (6)	1.000
Spondylitis	7 (5)	1 (6)	1.000
Arthritis, all	10 (7)	2 (12)	0.620
Periprosthetic joint infection	6 (4)	0	NA
Pneumonia	19 (13)	5 (29)	0.141
Empyema	1 (1)	0	NA
Endocarditis	5 (4)	0	NA
Aortitis	0	1 (6)	NA
Foreign body infection	3 (2)	0	NA
Puerperal sepsis	4 (3)	0	NA
Intraabdominal infection	2 (1)	0	NA
Endophthalmitis	2 (1)	0	NA
Bacteremia without defined focus	14 (10)	3 (18)	0.397

*Values are no. (%) except as indicated. Severe disease was defined as treatment in the intensive care unit or death within 30 days after hospital admission. NA, not applicable. †One patient might have >1 clinical manifestations.

Intravenous antimicrobial drugs were administered as the initial treatment for all episodes. The initial antimicrobial drug treatment was second-generation cephalosporin for 73% of patients with nonsevere disease and 47% of patients with severe disease. In 35% of patients with severe disease, the initial antimicrobial drug treatment was ceftriaxone. The initial antimicrobial drug treatments were effective for all episodes. After 2 days, the antimicrobial drug treatment was narrowed to ﻿penicillin G for 43% of all patients: 24% of those patients had severe disease, and 46% had nonsevere disease. Clindamycin treatment was provided to 29% of patients with severe disease and 10% of patients with nonsevere disease. No association was observed between clindamycin treatment and death rate. The median length of intravenous antimicrobial drug treatment was 10 (range 0–45) days. None of the patients received intravenous immunoglobulin therapy. All SDSE isolates were susceptible to ﻿penicillin and cephalosporins. Clindamycin resistance was found in 4 (3%) isolates. Erythromycin resistance was found in 16% of isolates, and 3% of isolates had intermediate sensitivity to erythromycin.

The 30-day case-fatality rate for all patients was 6% (9 patients). The 2-day case-fatality rate was 3%, and the 7-day case-fatality rate was 4%. The median age of deceased patients was 77 (range 69–88) years; 56% were male and 44% female. Associations between clinical characteristics and risk for death were studied (Table 2); 50% of all patients (48% of survivors and 78% of nonsurvivors [p = 0.094]) had >1 clinical characteristic significantly associated with risk factors for death, which included admission to an ICU, hypotension, need for vasopressors, lowered level of consciousness, septic shock, multiorgan failure, or surgical intervention. The case-fatality rates were high for patients who experienced unconsciousness (43%), multiorgan failure (33%), and septic shock (28%). Surgical intervention was needed for 24% of all patients. The most commonly required surgical interventions were amputation (5%), abscess drainage (5%), joint aspiration or lavage (3%), and prosthetic joint replacement or removal (3%). Most (84%) patients showed their first symptoms within 24 hours before hospital admission. We did not observe a significant difference in the median duration of symptoms before hospital admission between nonsurvivors and survivors (p = 0.858). Among survivors, 17 (11%) patients had nosocomial infections (symptoms manifested after prior discharge from inpatient care or >48 hours after hospitalization). In contrast, all patients who died had community-acquired SDSE bacteremia (p = 0.599).

**Table 2:** Disease severity among 159 episodes of *Streptococcus dysgalactiae* subspecies *equisimilis* bacteremia during November 2015–November 2019, Finland*

Characteristic	Total, n = 159	Survivors, n = 150	Nonsurvivors, n = 9	p value
Admitted to intensive care unit	11 (7)	8 (5)	3 (33)	0.017
Needed mechanical ventilation	3 (2)	2 (1)	1 (11)	0.161
Needed continuous renal replacement therapy	1 (1)	1 (1)	0	NA
Needed hemodialysis	1 (1)	1 (1)	0	NA
Needed vasopressors	15 (9)	12 (8)	3 (33)	0.041
Lowered level of consciousness†	37 (23)	31 (21)	6 (67)	0.005
Unconscious	7 (4)	4 (3)	3 (33)	0.004
Hypotension‡	36 (23)	30 (20)	6 (67)	0.005
Septic shock§	18 (11)	13 (9)	5 (56)	0.001
Disseminated intravascular coagulation¶	6 (4)	5 (3)	1 (11)	0.299
Multiorgan failure#	12 (8)	8 (5)	4 (44)	0.002
Underwent surgical intervention	38 (24)	33 (22)	5 (56)	0.037

*Values are no. (%) except as indicated; p values compare survivors and nonsurvivors. NA, not applicable. †Lowered level of consciousness (unconscious or confusion) >1 time during the first 2 days after positive blood culture. ‡Systolic blood pressure <90 mm Hg >1 time during days 0–2 after positive blood culture. §Use of vasopressor and blood lactate level >2 mmol/L >1 time during days 0–2 after positive blood culture. ¶Platelet counts <100 × 10^9^/L. #Observed >3 concomitant organ failures.

We calculated PBS for each patient and found it was a significant predictor of death (Table 3; Figure). ROC analysis indicated PBS had an AUC of 0.775 (95% CI 0.555–0.995; p = 0.014). The optimal cutoff determined by the Youden index was PBS >3; PBS was strongly associated with 30-day mortality at this cutoff, having 78% sensitivity and 87% specificity (OR 22.84 [95% CI 4.41–118.22]; p<0.001). A PBS >4 was also associated with 30-day mortality but had 44% sensitivity and 95% specificity (OR 15.54 [95% CI 3.41–70.96]; p = 0.002).

**Table 3:** Variables included in the Pitt bacteremia score in study of clinical aspects and disease severity of *Streptococcus dysgalactiae* subspecies *equisimilis* bacteremia, Finland*

Variable	Point allocation
Hypotension†	2
Mechanical ventilation	2
Mental status	
Disoriented	1
Unconscious	4
Body temperature, °C	
35.1–36 or 39–39.9	1
<35.0 or >40.0	2

*All variables recorded at hospital admission. †Systolic blood pressure <90 mm Hg or vasopressor required.

**Figure:**
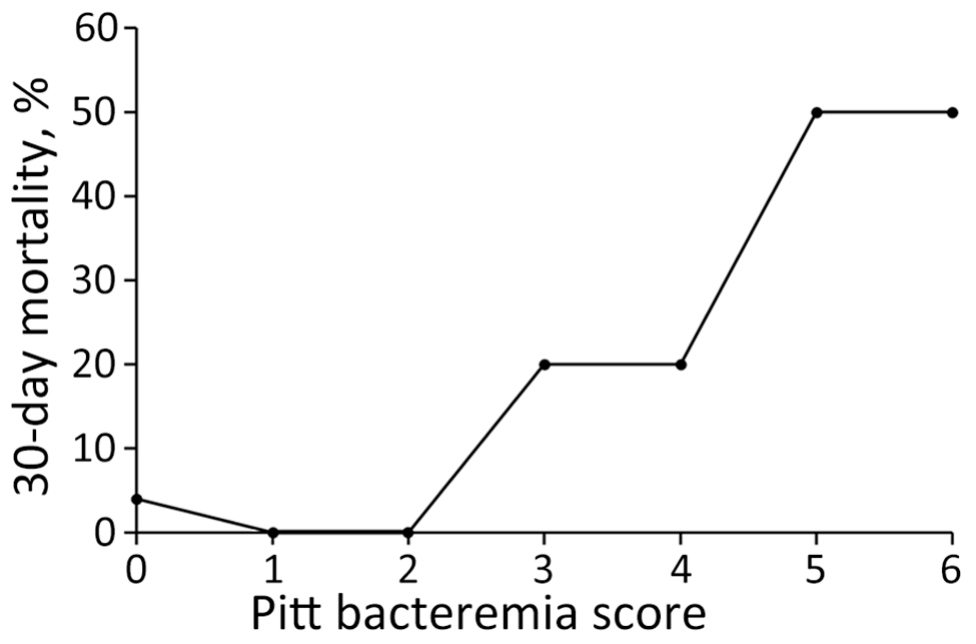
Association between 30-day mortality and Pitt bacteremia scores in study of clinical aspects and disease severity of *Streptococcus dysgalactiae* subspecies *equisimilis* bacteremia, Finland. Pitt bacteremia score was a significant predictor of death for infected patients with bacteremia.

Eleven patients with SDSE bacteremia were admitted to an ICU (Table 4). Their median age was 68 (range 35–85) years, and the median length of ICU stay was 3 (range 1–7) days; 2 patients were female and 9 male. Three of the 11 patients died, 10 manifested general deterioration, 9 had fever, and 6 had dyspnea. Cellulitis was the most common clinical manifestation in 5 patients, but several patients had >1 clinical sign. Eight patients were hypotensive and needed vasopressors, 3 patients needed mechanical ventilation, and 1 required continuous renal replacement therapy. All patients treated in an ICU had elevated blood lactate and procalcitonin levels. The median lactate level in blood was 3.5 (range 0.5–4.9) mmol/L; the median procalcitonin level in blood was 11.8 (range 2.1–88.5) µg/L.

**Table 4:** Clinical synopses of 11 patients with *Streptococcys dysgalactiae* subspecies *equisimilis* bacteremia who were treated in an intensive care unit during November 2015–November 2019, Finland

Patient no.	Age, y*/sex	Symptoms	Clinical syndrome	Continuous renal replacement therapy	Mechanical ventilation	Vasopressors	ICU stay, d	Outcome
1	80/M	Fever, general deterioration, dyspnea, cough	Cellulitis, pneumonia	No	No	No	3	Survived
2	60/F	Fever, general deterioration, dyspnea	Arthritis	No	Yes	No	3	Survived
3	71/M	Fever, pain, general deterioration, altered mental status, dyspnea	Cellulitis, pneumonia	No	No	Yes	3	Died
4	69/F	Fever, general deterioration, nausea, altered mental status, dyspnea	Purulent skin infection	No	No	Yes	4	Died
5	68/M	General deterioration, dyspnea	No defined focus	No	No	Yes	1	Survived
6	85/M	Fever, general deterioration, nausea, dyspnea	Cellulitis	No	No	Yes	1	Survived
7	43/M	General deterioration	Cellulitis, spondylitis, arthritis, osteomyelitis	No	No	Yes	2	Survived
8	59/M	Fever, general deterioration, altered mental status	Cellulitis	Yes	No	No	4	Survived
9	82/M	Fever, pain, general deterioration, altered mental status	Necrotizing fasciitis	No	Yes	Yes	2	Died
10	35/M	Fever, pain, nausea	Necrotizing fasciitis, abscess	No	No	Yes	2	Survived
11	66/M	Fever, general deterioration, nausea, altered mental status	Pneumonia	No	Yes	Yes	7	Survived

*Age at hospital admission.

We determined associations between laboratory test results from SDSE bacteremia patients and nonsevere or severe disease (Table 5). Decreases in leukocyte counts and C-reactive protein (CRP) levels in blood during the first days of treatment were less for patients with severe disease than those with nonsevere disease. The leukocyte counts were significantly higher on days 2 and 3, and CRP levels were higher on days 3 and 4 after admission in patients with severe disease than those with nonsevere disease. Blood creatinine levels were higher in patients with severe disease than those with nonsevere disease at admission and during the first day of hospitalization.

**Table 5:** Laboratory test results of patients with *Streptococcus dysgalactiae* subspecies *equisimilis* bacteremia in relation to nonsevere and severe disease during November 2015–November 2019, Finland*

Laboratory test	Nonsevere disease, n = 142	Severe disease, n = 17	p value
Leukocyte count, × 10^9^ cells/L			
Day 0	16 (11–19)	14 (11–21)	0.973
Day 1	13 (10–18)	15 (11–23)	0.370
Day 2	11 (8–15)	15 (10–21)	0.032
Day 3	10 (7–12)	15 (10–16)	0.020
Highest†	17 (12–21)	19 (12–25)	0.488
C-reactive protein, mg/L			
Day 0	70 (20–179)	196 (21–344)	0.093
Day 1	177 (123–274)	195 (94–360)	0.439
Day 2	207 (124–283)	256 (120–348)	0.329
Day 3	149 (79–217)	217 (124–305)	0.030
Day 4	99 (51–163)	193 (100–260)	0.009
Highest‡	237 (163–320)	328 (162–385)	0.202
Creatinine, µmol/L			
Day 0	94 (78–122)	113 (84–203)	0.046
Day 1	93 (81–121)	135 (89–182)	0.043
Day 2	94 (73–121)	103 (80–175)	0.137
Platelet count, × 10^9^/L			
Day 0	196 (159–234)	169 (143–216)	0.178
Day 2	183 (146–217)	142 (105–212)	0.175
Highest ALT,§ U/L	26 (18–41)	58 (25–69)	0.052

*Values are median (interquartile range) except as indicated. Severe disease was defined as treatment in the intensive care unit or death within 30 days after hospital admission. ALT, alanine aminotransferase. †Highest count during days 0–3. ‡Highest level during days 0–4. §Highest level during days 0–4.

We analyzed the predictive value of laboratory test results for severe disease by using ROC curves. The leukocyte counts on days 2 and 3, the CRP levels on days 3 and 4, and the highest level of alanine aminotransferase (ALT) during days 0–4 after admission were significant predictors of severe disease. The AUC for leukocyte counts was 0.680 (95% CI 0.522–0.839; p = 0.026) on day 2 and 0.713 (95% CI 0.584–0.877; p = 0.011) on day 3. The AUC for CRP was 0.697 (95% CI 0.553–8.62; p = 0.019) on day 3 and 0.740 (95% CI 0.569–0.991; p = 0.006) on day 4. The AUC of the highest ALT level during days 0–4 was 0.674 (95% CI 0.501–0.848; p = 0.049).

We determined optimal cutoff levels for laboratory test results predicting severe disease by using the Youden index. A leukocyte count >14.1 × 10^9^ cells/L on day 2 had a 64% sensitivity and 74% specificity (OR 4.48 [95% CI 1.37–14.61]; p = 0.021). A leukocyte count >12.5 × 10^9^ cells/L on day 3 had a 64% sensitivity and 80% specificity (OR 6.78 [95% CI 1.83–25.08]; p = 0.004). CRP levels >199 mg/L on day 3 had a 64% sensitivity and 72% specificity (OR 4.50 [95% CI 1.24–16.33]; p = 0.035). CRP levels >163 mg/L on day 4 had a 73% sensitivity and 75% specificity (OR 8.00 [95% CI 1.97–32.42]; p = 0.002). The highest measured ALT level during days 0–4 that was >53 U/L had a 67% sensitivity and 79% specificity (OR 7.65 [95% CI 2.06–28.44]; p = 0.002).

## Discussion

We observed an association between cellulitis and nonsevere disease in SDSE, whereas necrotizing fasciitis was associated with severe disease. A previous study also indicated that cellulitis, which is a clinical manifestation of β-hemolytic streptococcal bacteremia, predicted a favorable prognosis (*8*). In this study, 3 of 4 patients with necrotizing fasciitis had severe disease, and 2 of those patients died. Mortality rates of 19%–33% have been reported for patients with necrotizing fasciitis caused by SDSE (*10*,*18*). Endocarditis caused by SDSE has a high prevalence of embolic complications and high mortality rates, similar to that for endocarditis caused by *Staphylococus aureus* (*16*,*17*,*23*). Our findings diverge from those previous studies; all 5 patients with endocarditis had nonsevere disease, and none experienced embolic complications or required surgical intervention. Pneumonia seemed to be associated with severe disease, although statistical significance was not reached. No data on the association between pneumonia and death in patients with SDSE bacteremia are available, but pneumonia has been associated with marked mortality rates for patients with group A *Streptococcus* bacteremia (*24*,*25*). In contrast to previous studies, patients without a defined disease focus had relatively favorable outcomes.

In our study, 50% of patients manifested >1 clinical characteristic associated with risk for death. Despite this finding, the case-fatality rate (6%) remained relatively low. In previous studies of SDSE bacteremia, 6%–24% of patients required treatment in an ICU, 7%–19% required vasopressors, and 0%–17% required ventilation support (*12*–*14*). In another study, 70% of SDSE episodes were associated with markers of severe disease, including ICU admission and the need for vasopressors or ventilation support (*11*).

Surgical intervention was required for 24% of all SDSE bacteremia patients and 55% of patients treated in an ICU. In previous studies on SDSE bacteremia, the reported need for surgical intervention showed substantial variability, ranging from 18% to as high as 58.8% (*11*,*12*,*26*,*27*). For patients with group A *Streptococcus* bacteremia, surgical intervention was required in 37% of patients overall (*28*) and 61% of patients treated in an ICU (*24*).

Case-fatality rates ranging from 2% to 21% have been reported for patients with SDSE bacteremia (*12*,*29*), but no consistent trend in case-fatality rates has been identified. However, in a previous study from the Pirkanmaa health district, the case-fatality rates were 22% for patients with group C and 15% for those with group G *Streptococcus* bacteremia (*8*). For all bloodstream infections in Finland, case-fatality rates have remained stable at 13% since 2004 (*30*). The relatively low case-fatality rates in our study could be partly attributed to early recognition of the disease and enhanced treatment protocols.

In this study, we showed a clinical overview of ICU-treated SDSE bacteremia. Most patients admitted to an ICU exhibited nonspecific symptoms, such as general deterioration, fever, and dyspnea. Among the patients treated in an ICU, 2 had necrotizing fasciitis, which was identified as a risk factor for severe disease. However, most ICU-treated patients manifested nonnecrotizing soft tissue infections or pneumonia. Although data on ICU treatment for patients with SDSE bacteremia is lacking, studies on ICU-treated group A *Streptococcus* bacteremia indicated cellulitis and pneumonia were the most common clinical manifestations (*24**,**25*). However, in ICU-treated group A *Streptococcus* bacteremia, the proportion of necrotizing fasciitis cases has been substantially higher than that for SDSE bacteremia (*24*,*31*). In our study, most ICU-treated patients survived, possibly because of effective treatment but also careful patient selection practice, which excluded those with a very poor prognosis. Furthermore, TAUH has high-dependency units, where treatments such as vasopressor support are administered, lowering the need for ICU treatment.

The PBS has been used for >30 years to assess mortality risk for bloodstream infections (*32*). Our findings indicate that PBS serves as an accurate predictor of death from SDSE bacteremia. Although the previously validated cutoff value of >4 had high specificity in our study, it was achieved at the expense of sensitivity. In contrast to previous studies, we identified an optimal PBS cutoff point of >3, which achieved a maximum balance between sensitivity and specificity. This deviation from previous findings might be attributed to the relatively small number of patients in our study who died. In addition, excluding cardiac arrest from the score might have partly contributed to the relatively low overall scores.

We observed erythromycin resistance in 16% and clindamycin resistance in 3% of SDSE isolates. Increasing SDSE resistance to erythromycin or clindamycin have been reported. In Norway, resistance to both clindamycin and erythromycin, which was absent before 2009, increased to 12% during 2016–2018 (*33*). Similarly, in Japan, macrolide resistance increased from 10.3% during 2003–2005 to 18.5% during 2010–2013 (*4*,*34*).

We did not observe significant associations between leukocyte counts or CRP levels and disease severity at admission. However, significant associations with severe disease were identified on days 2 and 3 for leukocyte counts and on days 3 and 4 for CRP levels after admission. In an SDSE bacteremia study in Japan, an insufficient leukocyte response and thrombocytopenia at admission were linked to poor outcomes, but CRP levels were not predictive at admission, which agrees with our findings (*13*). Confirming our findings, another study on the association between CRP levels and death in patients with sepsis showed that a CRP value >100 mg/dL on day 3 after hospital admission was linked to a fatal outcome (*35*). Our findings suggest that monitoring leukocyte and CRP responses during the initial days of treatment is crucial. If an adequate response is not observed after a few days of treatment, heightened attention to more intensive treatment is warranted.

A major strength of this study is the prospective design, enabling the systematic and reliable collection of a broad range of clinical data. All data were consistently gathered by the same infectious disease specialist, and the study encompassed a relatively large population. However, some data used to determine PBS in previous studies were unavailable in our study. Although scoring performed well in predicting death, limited available data might have contributed to the comparatively lower scores observed in our study. Data collected from laboratory test results were also limited.

In conclusion, this prospective study on SDSE bacteremia revealed necrotizing fasciitis is a risk factor for severe disease, whereas cellulitis is associated with a favorable prognosis. Despite a substantial proportion of patients manifesting characteristics associated with mortality risk, the overall mortality rate remained relatively low, underscoring the importance of prompt and effective treatment. The PBS accurately predicted death of patients with SDSE bacteremia. Leukocyte counts and CRP responses during the initial days of treatment emerged as valuable indicators of disease severity. Clinicians should consider using the PBS, leukocyte counts, and CRP responses during initial treatment with antimicrobial drugs to improve treatment strategies and survival of patients with SDSE.
